# The Mechanism and Latest Research Progress of Blood–Brain Barrier Breakthrough

**DOI:** 10.3390/biomedicines12102302

**Published:** 2024-10-10

**Authors:** Fei Wang, Liujie Qi, Zhongna Zhang, Huimin Duan, Yanchao Wang, Kun Zhang, Jingan Li

**Affiliations:** 1 School of Materials Science and Engineering, Zhengzhou University, Zhengzhou 450001, China; wangfei9717@gs.zzu.edu.cn (F.W.); qiliujie@gs.zzu.edu.cn (L.Q.); zhongna_zhang@gs.zzu.edu.cn (Z.Z.); duanhuimin@gs.zzu.edu.cn (H.D.); wangyancaho@gs.zzu.edu.cn (Y.W.); 2 School of Life Science, Zhengzhou University, Zhengzhou 450001, China

**Keywords:** cerebrovascular disease, blood–brain barrier, passive diffusion, receptor-mediated transport, adsorption-mediated transport, carrier-mediated transport

## Abstract

The bloodstream and the central nervous system (CNS) are separated by the blood–brain barrier (BBB), an intricate network of blood vessels. Its main role is to regulate the environment within the brain. The primary obstacle for drugs to enter the CNS is the low permeability of the BBB, presenting a significant hurdle in treating brain disorders. In recent years, significant advancements have been made in researching methods to breach the BBB. However, understanding how to penetrate the BBB is essential for researching drug delivery techniques. Therefore, this article reviews the methods and mechanisms for breaking through the BBB, as well as the current research progress on this mechanism.

## 1. Introduction

Cerebrovascular disease encompasses a range of brain disorders resulting from damage to the vasculature and blood supply. This includes various pathological conditions such as stroke, bleeding, and other issues related to blood vessel narrowing, blockage, rupture, and inflammation. A major contributing factor to dementia and stroke, both of which call for long-term care, is cerebrovascular disease. Numerous pathophysiological processes are involved in these diseases, and one of the main drivers of inflammation, apoptosis, and oxidative stress is mitochondrial dysfunction. These processes damage neurovascular units by inducing glial cell activation, endothelial cell death, neuronal cell death, and disruption of the BBB [1]. Hemorrhagic transformation following ischemic stroke typically occurs before the breakdown of the BBB. BBB permeability increases within the first few hours after a stroke, potentially leading to the progression of ischemic tissue damage, secondary neuroinflammation, vasogenic edema formation, and cerebral hemorrhage [2]. BBB permeability can be restored with prompt reperfusion. However, the release of proteases such as matrix metalloproteinases (MMP) and the generation of reactive oxygen species (ROS) by infiltrating white blood cells worsen BBB destruction after late reperfusion [2,3]. This indicates that the course of spontaneous or therapy-induced reperfusion following a stroke depends critically on the timing and degree of BBB breakdown. This indicates that the course of spontaneous or therapy-induced reperfusion following a stroke depends critically on the timing and degree of BBB breakdown. Therefore, choosing a course of treatment following an acute ischemic stroke may benefit from an understanding of the precise processes of BBB transmission.

**Figure 1:**
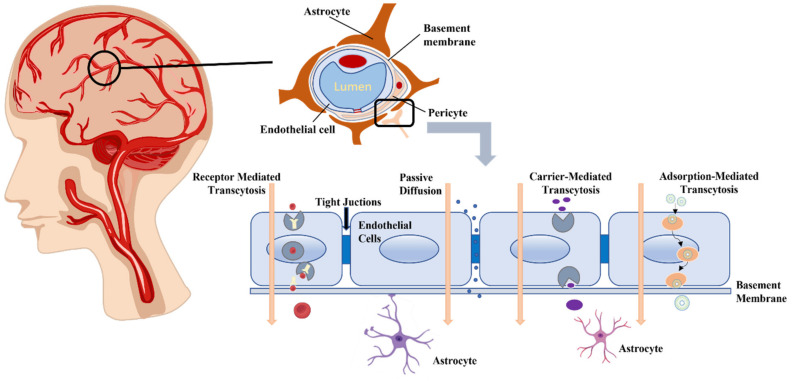
The structure of BBB and four methods of breaking through BBB.

The bloodstream and the CNS are separated by the intricate blood vessel network known as the BBB. Its main role is to regulate the environment within the brain [4]. Its unique multicellular structures control this barrier’s primary functions, which include preserving brain homeostasis, regulating the transfer from the inside to the outside, and providing protection from injury. Each kind of cell significantly contributes to maintaining the integrity of the BBB. In addition to serving as a barrier, it actively regulates the flow of substances into and out of the blood–brain interface. The BBB establishes an environment that supports proper neuronal function by regulating the movement of molecules and ions, providing neurons with necessary nutrients and oxygen, and safeguarding the brain from toxins and pathogens. However, the functional filtration mechanism of the BBB makes it difficult for almost all macromolecules and 98% of small molecules to enter brain tissue from the blood [5]. The primary obstacle for drugs to enter the CNS is the low permeability of the BBB [6], presenting a significant hurdle in treating brain disorders. The review will begin with an overview of the BBB’s structure and function, then outline four approaches to effectively bypassing this barrier (as depicted in Figure 1) and examine the mechanisms of crossing as well as recent advancements in research.

## 2. Physiological Basis of the BBB

### 2.1. The Structure of BBB

The BBB is a lipid membrane with an “effective” pore size of only 1.4~1.8 nm at the tight junctions of capillary endothelial cells. Because of the lipophilic nature and morphological structure of the membrane, small lipid-soluble substances from the peripheral blood vessels can directly pass through and fuse with the lipid membrane to enter the brain, and small water-soluble drugs can also diffuse through the cellular spaces on the membrane surface to enter the brain. Water-soluble drugs of small molecular size are able to directly enter the brain through the intercellular space on the membrane surface, while larger molecule substances like proteins, peptides, and gene drugs cannot pass through the BBB via passive diffusion due to the tight junctions (TJs) of neuroglial material between endothelial cells (ECs) of brain capillaries, which are negatively charged. Hence, drugs with a positive charge or those that are electrically neutral are more likely to penetrate the BBB than those with a negative charge. The BBB functions as a critical checkpoint for brain access and regulates the internal environment of the CNS through TJs between ECs, astrocytic end-feet, and the basement membrane (BM) [7,8]. With its low permeability and strong selective hindrance, the BBB serves as a dynamic barrier that separates peripheral blood from neuronal cells in the brain and spinal cord, which is essential for preserving CNS homeostasis. Brain microvascular endothelial cells (BMECs) and TJs comprise the innermost layer of the BBB, followed by BM and pericytes in the middle layer and astrocytes and extracellular matrix in the outer layer. Among these components, ECs and TJs are fundamental structures in maintaining BBB integrity. The role of TJs is essential for inhibiting the transfer of ions and small hydrophilic solutes via the para-cellular route, effectively regulating their transportation [9,10,11,12]. Other important molecules are mainly transported into the brain through transcellular pathways, usually via active transporters.

#### 2.1.1. Endothelial Cells

ECs are a thin layer of flat cells that are in the medial interdigitated vasculature and are the first physiological barrier to the BBB, capable of limiting trans (cross-cell) and intercellular (paracellular) transport [13]. Unlike ECs in other tissues, brain capillary cells are linked to each other by TJs, which effectively prevent the passage of most molecules and restrict the accumulation of hydrophilic molecules. Although lipid-soluble substances with low molecular weight can pass through the BBB via diffusion, water-soluble substances like glucose and amino acids cannot permeate it. The BBB possesses mechanisms to facilitate the transportation of glucose and essential amino acids [14]. Active efflux systems eliminate compounds that accumulate in the brain, while they also express proteins within endothelial cells and at the boundaries between endothelial cells, thus preventing cells from paracrine transport. However, in the event of inflammation, the permeability of ECs is enhanced, which may be caused by a local loss of connections between microvascular endothelial cells due to altered cellular contractility or the opening of intercellular junctions. These proteins can be classified into three main groups: TJs, adherens junctions (AJs), and cell adhesion molecules (CAMs) [15]. The intercellular gaps between cells that form TJs range from 10 to 20 nm and overlap each other, with the TJs between ECs being 50–100 times tighter than those of the surrounding capillary endothelium. TJs are made up of complex proteins, such as cytoskeletal, transmembrane, and cytoplasmic adhesion proteins, that bridge the gap between cells [16]. Claudins, junction adhesion molecules (JAMs), and occludin proteins are examples of transmembrane proteins [17]. The three forms of Cingulin that make up the cytoplasmic adhesion proteins are ZO-1, ZO-2, and ZO-3. The BBB’s ability to function is directly correlated with the expression and arrangement of these proteins. The expression of TJ-related proteins may be impacted by specific medications or pre-treatments, which may therefore have an effect on the BBB’s permeability. Additionally, ECs indirectly control the brain’s physiological processes. The BBB can be crossed by metabolic byproducts from glucose within the endothelium, and once within the brain, they are transformed into neurotransmitters [16].

#### 2.1.2. Basement Membrane

The BBB is surrounded by BM, which is a complex network of extracellular matrix proteins that sits on the lower surface of the BMEC and is typically between 20 and 60 nm thick [18]. The BM consists of a continuous meshwork of collagen, laminin, and various protein fibers such as fibronectin, which provide binding sites for signal transduction of various cytokines including vascular endothelial growth factor (VEGF), norrin, Wnt, etc., or molecules and cells external to the neural tissue that require passage through the barrier. Additionally, it anchors cellular components to the barrier through cell type-specific integrin and dystrophin receptor interactions [19].

#### 2.1.3. Pericytes

Pericytes are morphologically diverse motile cells that are distributed in the basement membrane of the cerebral microvascular system and are essential for maintaining endothelial cell-specific gene expression in the BBB. By releasing various growth factors and angiogenesis factors, they regulate vascular permeability. They can also move to the site of injury and build new blood vessels. Pericytes can enter the neural tissue after basement membrane rupture and become macrophages, thus providing a barrier to systemic circulation.

#### 2.1.4. Astrocytes

Astrocytes are a significant constituent of the BBB and neurovascular unit, and are capable of regulating the stability and acid–base equilibrium of extracellular ions in the CNS. They are highly polarized cells located around the microvasculature, extending the perivascular end-foot and wrapping the endothelium. A secondary barrier called the glial limiting factor, which is the end foot that wraps around the synapse, is formed, where they regulate, receive, and are directly involved in synaptic signaling through neuro glial transmission [20]. Not only do astrocytes further support neuronal metabolism by storing and supplying metabolites as needed, they also play a key role in removing brain waste.

### 2.2. The Discovery of BBB

In the late 1600s, Humphrey Ridley described the first concept of the special protective properties of the brain. Additionally, in the early 20th century, Lewandowki carried out experiments on animals and suggested that brain capillaries selectively limit the passage of specific molecules [9]. More than a century ago, Paul Ehrlich identified the BBB that separates the blood circulation from the CNS, and he administered aniline dye, which is water-soluble, into the outer regions of animal organs to facilitate quick distribution and flow throughout the body. However, this method was not successful in reaching the brain and cerebrospinal fluid. Ehrlich explained these observations by suggesting that the dye did not have a strong affinity for the endothelium of the cerebrovascular system. Shortly after, Edwin E. Goldman, a student of Ehrlich, discovered that certain dyes injected into the cerebrospinal fluid could selectively stain neural tissue [21], indicating their inability to enter the blood circulation of the brain. In the same year, he initially proposed the concept of a vascular BBB, also known as the BBB, based on his hypothesis. Subsequently, there was increasing recognition of the BBB [22]. The observation of membrane barriers was not made until 1937, with the invention and widespread use of the scanning electron microscope (SEM) in medical research. A variety of drug delivery systems (DDS) have been created, including inorganic and organic nanocarriers. In addition, inorganic materials are more commonly used for imaging than carriers. In contrast, organic nanocarriers offer the potential for drug delivery across the BBB due to their biocompatibility, low toxicity, and ease of modification and functionalization. In 2018, Furtado and colleagues conducted a comprehensive analysis of the structure, function, and disorders related to the BBB, as well as provided a summary of strategies for circumventing or penetrating the BBB. However, only a few sections of the article described the use of nanocarriers to cross the BBB [23,24,25]. In 2019, Xie et al. [26] reviewed various methods of delivering substances across the BBB, including intranasal delivery, temporary blockade of the BBB, local delivery, and receptor- or peptide-mediated strategies for crossing the BBB.

### 2.3. Function of the BBB

The brain contains tens of billions of neurons that collaborate to sustain the body’s normal functioning, but these neurons are extremely fragile and need to be in a highly stable and strictly controlled internal environment. The main function of the BBB is to block the entry of external substances (such as microorganisms, toxins, inflammatory factors, and antibodies) into the brain through the bloodstream. The purpose of this is to minimize or avoid potential harm to the brain from these toxic substances in the bloodstream and uphold a consistent internal environment in the brain.

As for the characteristics of the BBB, the first is protective. The BBB can regulate body fluids and protect the brain. Cerebral brain diseases such as tumors, inflammation, and injury can increase the permeability of the BBB, leading to the disappearance of its function [27]. Some of the original substance cannot enter the brain, and the substance can randomly travel in and out of the nervous system, leading to the patient’s discomfort. We must consult a doctor promptly to protect the brain function. The second factor is the solutes’ affinity for lipids in the bloodstream, which determines their capacity to permeate brain tissue via the ECs of brain capillaries. The ease and speed at which solutes can pass through this barrier depend on their level of solubility in lipids present in the blood. According to this rule, we can enhance certain CNS drugs to facilitate their entry into brain tissue and expedite drug release. According to this principle, certain CNS medications can be enhanced to facilitate penetration of the BBB and expedite drug efficacy. For example, barbiturates are a kind of central anesthetic, but they have weak lipophilicity and enter the brain tissue slowly, so they can be improved to phenobarbital with stronger lipophilicity, which can allow them to more easily enter the brain tissue through the BBB and play the role of hypnotic anesthesia very quickly. The third is small molecules. Small molecules solute penetrate the BBB by passive diffusion and must have high lipophilicity at the same time, with low electronegativity and other characteristics. When solutes are dissolved in water, they have the ability to engage with the oxygen atoms found in water molecules, forming hydrogen bonds irrespective of their positive or negative charge. Higher solubility and decreased BBB permeability are directly correlated with a solute’s capacity to form hydrogen bonds. Water has a much smaller molecular weight compared to glucose and other solutes, allowing it to pass through endothelial cells and astrocytes connecting different parts of the brain. Adrenaline and norepinephrine, due to their strong solubility in water and high hydroxy group content, have difficulty crossing the BBB. Fourthly, in terms of carrier operation, BMECs are equipped with abundant carrier proteins that facilitate the transportation of substances from the blood to the ECs through the BBB at a slower speed, such as glucose, which serves as the primary energy source for brain tissue metabolism. But with the help of glucose carriers, they are able to pass through the BBB very quickly in order to promptly fulfill the requirements of brain metabolism. Recognized carriers currently consist of hexose carriers, neutral amino acid carriers, basic amino acid carriers, and short-chained single-carboxylic acid carriers.

## 3. Methods of BBB Breach

The BBB prevents many large molecules from entering the brain. Only small molecules and lipid-soluble compounds that satisfy specific requirements, such as having a molecular weight of less than 500 Da, fewer than five hydrogen bond donors, fewer than ten hydrogen bond acceptors, and an octanol-water partition coefficient of less than or equal to five, can pass through the BBB [28]. In addition to impeding the passage of large molecules into the brain, the blood–brain barrier (BBB) also restricts the uptake of over 98% of tiny molecules, including cancer therapy medications like vincristine and methotrexate [29]. Essential molecules and therapeutic drugs can utilize multiple pathways to cross the BBB, including passive diffusion, receptor-mediated transport (RMT), adsorption-mediated transport (AMT), and carrier-mediated transport (CMT) [30]. In addition to these strategies, there are physical ways to deliver drugs across the blood–brain barrier to the brain, such as: low-intensity pulsed ultrasound (LIPU)-mediated transport, magnetic-field-enhanced permeation, electromagnetic field, and electroporation.

### 3.1. Passive Diffusion

The most direct route of transcellular transport is passive diffusion [24,31]. The passage of ions and small molecules across a membrane due to variations in concentration and potential without the need for a particular transport medium or carrier is known as passive diffusion [32]. Passive diffusion is significantly restricted by the unique structure of the BBB. Thus, only low-molecular-weight, non-dissociative, lipophilic molecules can more easily cross the endothelial membrane through passive diffusion across the BBB, while other molecules must enter through other routes [33]. While passive diffusion may not be the predominant method for drugs to traverse the BBB, it is likely to be an auxiliary process to increase the transport speed and percentage of drugs. Kiyohiko investigated the mechanisms involved in the transportation of drugs, including passive diffusion and CMT [34]. RMT and passive transport mechanisms were both observed in drug absorption experiments conducted on intestinal epithelial cells.

Passive diffusion allows small, lipophilic substances like carbon dioxide to diffuse across the BBB [35]. BBB permeability is influenced by a number of physiological factors, such as cerebral blood flow, enzymatic activity, plasma protein binding, and efflux transporters like P-glycoprotein (P-gp) [35,36].

In recent years, clinical studies have demonstrated the favorable BBB permeability of mannitol and aromatic substances. Mannitol, the most commonly used penetrant, induces hypertonic contraction of ECs and subsequently opens the TJs of ECs [37], thus promoting passive diffusion of macromolecules across the BBB. However, opening the BBB non-selectively can lead to unregulated entry of both low- and high-molecular-weight substances, as well as an increase in brain fluid. This can result in neurotoxicity, aphasia, and hemiparesis, which pose potential risks to patient safety. Long-term evaluation of mannitol is necessary to determine its clinical value and suitability for use. In the study by Chunggab Choi et al. [38], the co-administration of mannitol and temozolomide (TMZ) was shown to decrease TJ proteins, which led to an elevation in the permeability of the BBB.

Out of these fragrant compounds, bergamot (BO) is a basic bicyclic monoterpene, exhibits favorable properties for enhancing drug penetration through the BBB, and is commonly utilized in traditional Chinese medicine. Shen et al. [39] used phacoemulsification to create a range of vinpocetine nanoemulsions with varying amounts of Bo. Bo dramatically improves the distribution of medications in the hippocampus and cortex following intranasal delivery in Alzheimer’s patients, according to quantitative research and in vitro imaging investigations. Liu et al. [40] used the thin film dispersion method to synthesize BO-modified DTX plus tetramine micelles, and then they conducted in vitro and in vivo experiments to examine the drug’s potential for treating gliomas. In vitro, these micelles aid in the drug’s BBB crossing by increasing bEnd.3 absorption. Targeting micelles encourages apoptosis and suppresses cell division. Consequently, BO is regarded as a promising and effective agent for facilitating drug delivery across the BBB.

### 3.2. Receptor Mediated BBB Crossing

Delivery of a variety of endogenous macromolecules into the brain relies on cellular transport pathways mediated by receptors. Through endocytosis, the binding of ligands to their specific receptors on the surface of the BMEC facilitates the formation of vesicles [41], and then the corpuscles formed pass through the BBB and enter the CNS through exocytosis to release ligands, thus exerting its biological functions. This process is referred to as RMT for BBB crossing [42]. Various receptor ligands can be attached to nanomaterials in order to enhance the transport of therapeutic agents into the CNS through the corresponding receptor-mediated BBB crossing pathway [43], which is a promising BBB-crossing strategy in the biomedicine field.

#### 3.2.1. Transferrin Receptor

**Figure 2:**
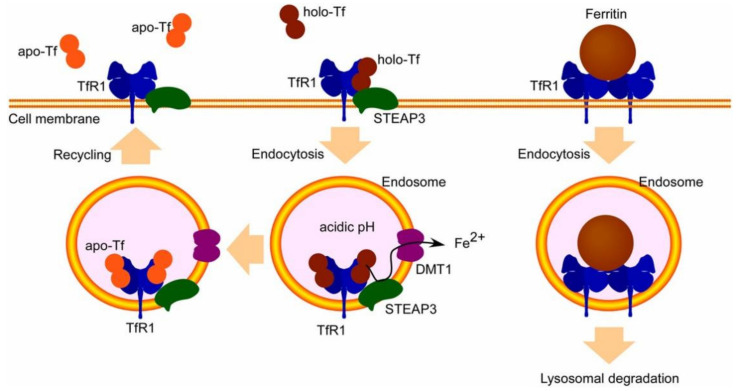
Transferrin receptors 1 (TfR1) facilitate the absorption of transferrin (Tf) and ferritin. Specifically, iron-saturated total Tf attaches to TfR1 on the surface of cells, and this combination is then taken into the cell through clathrin-mediated endocytosis [50].

One of the RMT receptors that has been investigated in great detail is the transferrin receptor (TfR), which is involved in the delivery of iron to the brain [44]. The release of Fe^3+^ from Tf occurs in the acidic conditions of the endosome, where it is then transformed into Fe^2+^ by metal reductase. Afterward, divalent metal transporter 1 (DMT1) transports it to the cytosol. The Tf/TfR1 complex in the endosome is then transported to the cell surface within circulating endosomes, where apo-Tf is released into the bloodstream (Figure 2). TfR exhibits high expression on brain endothelial cells (BECs) and possesses a strong affinity for Tf [45]. Tf-conjugated NPs can avoid uptake by the reticuloendothelial system (RES) through polyethylene glycol (PEG) surface modification [46]. A case in point is the application of Tf in conjunction with PEG NPs to augment the targeted delivery of Rhodamine 6G to the striatum, brain, and third ventricle in albino rats. The growth inhibition impact on cancer cell lines when temozolomide was loaded exceeded 70% [47]. Compared to unmodified micelles, the BECs absorption of Tf-anchored micelles loaded with c [RGDfK]-paclitaxel coupling compound (c [RGDfK] is a peptide specifically bound to integrin) was significantly increased [48]. Moreover, RI7217 is a monoclonal antibody directed against TfR. The antibody has been thoroughly investigated and has demonstrated promise for both medication delivery and treatment. Ultrasonic BBB penetration was achieved by Dasgupta et al. [49] using non-spherical polyacrylonitrile-butadiene-styrene microbubbles treated with RI7217. Actively targeted non-spherical microvesicles bind endothelial cells significantly more efficiently than spherical microvesicles, according to both in vitro and in vivo investigations. In conjunction with transcranial focused ultrasound, they markedly improved the BBB opening and the buildup of model medications.

#### 3.2.2. Insulin Receptor

The insulin receptor (IR), a transmembrane glycoprotein made up of two β and two α chains joined by disulfide bonds, has been the focus of much research as a component of the RMT system, helping to transport insulin from the bloodstream to brain tissue [51,52].

The short duration of insulin in the body (10 min) and the risk of low blood sugar from external insulin use limit the practical application of insulin as a targeted carrier for RMT [53]. Additionally, the monoclonal antibodies mAb83-14, mAb83-7, and mAb29B4 targeting mouse insulin receptor can be internalized and transported to the brain by endocytosis through binding to distinct epitopes of human insulin receptors α subunits [51]. The mAb29B4-modified HSA NPs are capable of crossing the BBB and demonstrating substantial anti-injury effects [54]. Compared to other antibodies, mAb83-14 is commonly utilized for brain delivery. Additionally, carriers modified with mAb83-14 can be employed for drug delivery as well.

#### 3.2.3. LDL Receptor

In the brain and other peripheral tissues, capillary ECs express the low-density lipoprotein receptor (LDLR) [55]. Low-density lipoproteins (LDL) that can bind to LDLR include cholesterol [56], tocopherols [57], and apolipoprotein (Apo). LDLR is located in the small fossa membrane [58] and can be transported to brain tissue through the RMT process [59]. Therefore, there is a lot of promise for the use of an RMT system based on LDLR for drug delivery to the brain.

#### 3.2.4. Lactoferrin Receptor

Numerous cell types have been found to contain the lactoferrin (Lf) receptor (105 kDa), including hepatocytes, monocytes, lymphocytes, and breast epithelial cells [60]. In order to aid in the transport of Lf, the BCEC also have receptors for Lf with two distinct types of binding sites (high affinity: dissociation constant (Kd) = 6.67 nM, low affinity: Kd = 4815 nM) [61]. Lf is a glycoprotein that is associated with the transferrin family and has a molecular weight of 80 kDa. It is linked to iron cations. It goes through the BBB by means of endocytosis, which is mediated by the Lf receptor and may involve LRP (low-density lipoprotein receptor-related protein) [62].

### 3.3. Carrier-Mediated Transport

CMT is also known as allosteric diffusion, and it is a form of transport that occurs through specialized carriers in the membrane. CMT does not require energy, but is saturable and highly specific [63]. The potential of nanotechnology in conjunction with CMT is found in its capacity to improve medication administration efficacy, enable precise drug delivery to the brain, and increase BBB penetration. There have been many previous reports of CMT, but most of them have been related to cell-mediated immune responses and their detrimental effects on the human body [64].

A range of specific cells from the organism, including nanoparticles for cell-mediated drug delivery, have become one of the main subjects of research [65]. Monocytes, macrophages, and neutrophils have been used for CMT [66]. These cells can be acquired during the inflammatory stage of any CNS disorder, and they have a strong ability to communicate and travel to the brain’s inflammation site [67], where inflammation causes disruption of the BBB. The cellular uptake of nanoparticles and their ability to load onto cellular carriers are primarily influenced by the surface coating of the nanoparticles. Positively charged nanoparticles have been observed to demonstrate increased uptake by immune cells and stem cells in comparison to neutral nanoparticles. Positively charged nanoparticles, prepared by high pressure homogenization and loaded with antiretroviral drugs such as indinavir (IDV), ritonavir (RTV), and efavirenz (EFV) [68], demonstrate increased accumulation in mononuclear phagocytes (MP) compared to negatively charged nanocarriers. In recent years, monocyte-mediated macrophages were developed and showed good brain homing ability. Transport of superparamagnetic iron oxide nanoparticles SHP30 is mediated by monocytes, and good penetration can be obtained in inflamed brain regions.

Inorganic nanoparticles can be easily modified to carry drugs, improving drug targeting, durability, and controllability. This can effectively extend the drug’s half-life in the body, increase its distribution at tumor sites, and reduce toxicity and side effects. Drugs can be precisely delivered across the BBB with the use of inorganic nanomaterials, including magnetic iron oxide nanoparticles, zinc oxide, Au, and Ag. They are predominantly internalized by cells and lead to prolonged retention in the tumor mesenchyme as a result of enhanced permeability and retention (EPR).

#### 3.3.1. Au Nanoparticles (Au NPs)

Au NPs are metallic colloidal nanoparticles, the majority of which have a size smaller than 10 nm. Au NPs can encapsulate molecules of therapeutic value and can effectively enhance drugs through the BBB in neurodegenerative diseases. Au NPs are simple to prepare and can be prepared in controllable sizes [69], can be subjected to a wide range of surface modifications [70], and are biocompatible [71]. Various formats of Au NPs have been extensively used in the medical field due to their distinct physical and chemical properties [72]. These include uses in imaging, biosensing, photothermal therapy, microwave therapies, as well as delivering genes for timely cancer diagnosis and treatment or providing antitumor drugs [73]. At the same time, Au NPs do not compromise the integrity of the BBB when they traverse it. The ability of Au NPs to spontaneously penetrate the BBB in rats without the use of an external electric field was confirmed in experiments conducted by Hagit Sela et al. [74]. Liu et al. [75] discovered that resveratrol (Rsv) had a therapeutic impact on glioblastoma. As a result, Rsv-AuNPs were created by combining resveratrol with AuNPs. In vitro tests revealed that gold nanoparticle treatment dramatically reduced U87 cell migration, invasion, and proliferation. Subsequently, more animal and human trials on Rsv-AuNPs will be conducted before they are used in the clinical management of glioblastoma. Additionally, when examining how Au NPs penetrate the BBB mechanism, it was observed that the concentration of Au NPs in brain tissue across the BBB was halved when comparing the use of an ion channel blocker with a control group that did not utilize an ion channel blocker. Ion channel blockers such as dofetilide, phenytoin sodium, and verapamil have been demonstrated to regulate the permeation of Au NPs through the BBB. Conversely, these ion channel blockers impact the ability of Au NPs to penetrate the BBB by either directly reducing their passage through the channels or disrupting TJs.

#### 3.3.2. Silver Nanoparticles (Ag NPs)

Ag NPs are a type of metallic colloidal NPs commonly utilized in the manufacturing, biomedical, and engineering fields due to their exceptional antimicrobial activity. They typically range in size from 1 to 100 nm. Research in toxicology has demonstrated that Ag nanoparticles have the ability to penetrate the BBB and show a notable amount of buildup in the brain [76]. In a study by Samuel Salazar-García et al. [77], Ag NPs were found to induce damage to the BBB by increasing the extravasation of EB and decreasing the expression of claudin-5, which was associated with the overexpression of metallothioneins (MTs), which affect the body’s immune system, as evidenced by an increase in granulocytes. In addition to this, it has been shown that Ag NPs interact with brain microvasculature, causing BBB inflammation, astrocyte proliferation, and neuronal degeneration [78]. In a study by Lidia Strużyńska, Beata Dąbrowska-Bouta, and Grzegorz Sulkowski [79], the findings indicated that Ag NPs can increase the permeability of the BBB and that there is a potential toxic effect in the molecular mechanisms induced by Ag NPs mainly in the form of cellular stress, which can cause cell death. According to Ho-Chen Lin et al.’s study [80], Ag NPs accumulated more in mouse brain astrocytes (ALT) and mouse neuroblastoma nerve 2a (N2a) cells when the tight junction proteins claudin-5 and ZO-1 were disrupted in mouse BECs. Ag NPs also caused an increase in palmitic acid production in N2a cells. The generation of amyloid beta (Aβ) is stimulated by this production, and as a result, there is an increase in Aβ accumulation outside of nerve cells. This, in turn, triggers the release of inflammatory cytokines and eventually causes the death of N2a cells.

#### 3.3.3. Zinc Oxide Nanoparticles (ZnO NPs)

ZnO possesses some unique physicochemical properties [81] with a particle size ranging from 20 to 80 nm, making it extensively utilized in the areas of doping and catalysis. Numerous studies have indicated that the function of certain cells and tissues can be influenced by ZnO NPs [82]. However, it was not until Xie et al. [83] employed male Swiss mice as an animal model for depression to examine the specific impacts of ZnO treatment on the CNS, prompting their formal investigation into its effects on the BBB and the CNS. The results of the study showed that ZnO NPs had a significant ameliorative effect on specific behaviors as well as cognitive dysfunction in mice, which was achieved by promoting synaptic plasticity of neurons. In 2021, Liu et al. [84] prepared glutathione (GSH)-modified composite loaded genes of ZnO quantum dots (QDs), along with the transplantation of nerve growth factor (NGF), and it was utilized in the management of a model of Parkinson’s disease (PD). They developed a novel brain-targeted ZnO QDs gene delivery system (ZnO@Polymer-NpG), which has multiple functions such as neuroprotection and biomarker. In vitro and in vivo uptake of ZnO QDs complexes and gene release in a cytosolic mouse Parkinson’s disease model were tracked by fluorescence imaging. Fluorescence tracking results demonstrated that the ZnO QDs complexes could deliver genes across the BBB and subsequently release genes through lysosomal escape into the brain. Furthermore, both in-laboratory studies and live-animal experiments indicate that these ZnO QDs composites, carrying interfering genes and NGFs, have shown effectiveness in offering neuroprotection with minimal harm and halting the progression of neurodegenerative diseases in the treatment of PD models. A nanocomposite consisting of carboxymethyl cellulose, chitosan, and biocompatible polymers was presented by Shima Ostovar et al. [85]. ZnO NPs are added to this co-biopolymer to enhance its mechanical, chemical, and drug-loading capabilities. Graphene quantum dots (GQD) are one of them; they are utilized to enhance chemical characteristics and BBB penetration. The outcomes demonstrate the nanocomposite’s minimal cytotoxicity and strong anti-cancer capabilities.

#### 3.3.4. Magnetic Nanoparticles

**Figure 3:**
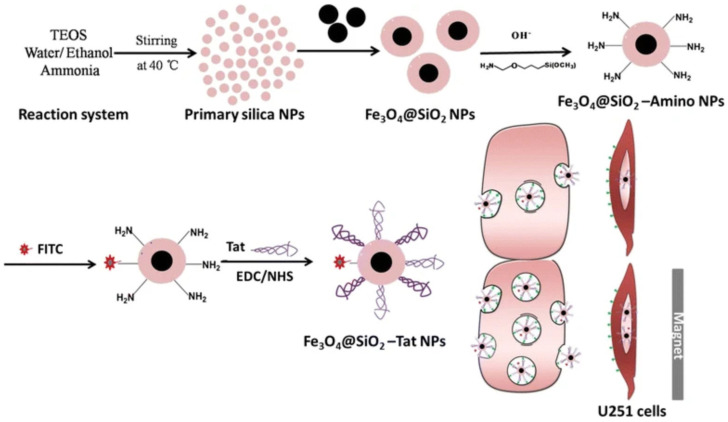
Design and synthesis of Fe_3_O_4_@SiO_2_-Tat magnetic NPs can enhance the permeability of the entire BBB [90].

Magnetic nanoparticles (MNPs) are magnetic properties characterized by their high surface-to-volume ratio and a propensity to agglomerate [86]. They are composed of magnetic cores encapsulated in organic or polymer coatings. The majority of MNPs are iron oxide nanoparticles (IONPs), which are prepared by magnetite (Fe_3_O_4_) and magnetic hematite (γ-Fe_2_O_3_) [87]. Because of their excellent biocompatibility and superparamagnetism, IONPs are widely used in the fields of targeted drug delivery, bio-imaging, thermotherapy, and photoablation therapy [88,89]. For example, Zhao et al. [90] developed a magnetic SiO_2_@Fe_3_O_4_ nanoparticle carrier, SiO_2_@Fe_3_O_4_-Tat (Figure 3), conjugated with the cell-penetrating peptide Tat. The uptake and localization of the nanoparticles in U251 cells were examined using a co-culture model of glioma cell U251 and human cerebral microvasculature EC (U251/HCMEC) to mimic the BBB. The research revealed that, following a 2 h addition to the magnetic field, there was a 17.4-fold rise in the absorption of SiO_2_@Fe_3_O_4_-Tat NPs by U251 cells. Additionally, it was noted that SiO_2_@Fe_3_O_4_-Tat NPs were able to pass through the BBB by crossing HCMEC and being pulled by a magnetic field. However, the toxicity of iron nanoparticles has also been widely concerning. When free Fe^2+^ reacts with hydrogen peroxide or oxygen, hydroxyl radicals and Fe^3+^ are generated, which triggers an oxidative stress reaction and in turn destroys molecules, such as DNA, finally leading to the obstruction of microcirculation in the organism’s organs and slow excretion, which may cause certain damaging effects. Hamed Nosrati, Mahsa Tarantash et al. [91] prepared IONPs@Asp using the BALB/c mouse model, and they formed two functional groups of functionalized nanoparticles on the surface of the nanoparticles, which were attached to paclitaxel (PTX), PEG, and glutathione (GSH), respectively. The analysis demonstrated its potential as a drug for MRI detection of PTX. Transcranial magnetic stimulation (TMS) was applied by Ye et al. [92] to enable the transport and delivery of MNPs to the brain parenchyma for fluorescent MNPs ranging in size from 50 to 300 nm. Rats under anesthesia were given MNP injections intraperitoneally (INP) and had TMS administered to the middle of their heads to test the theory. Consequently, compared to passive intranasal injection, TMS produced twice as much MNP delivery in the brain. Furthermore, a histological investigation was conducted to look into safety and showed that the major organs were not seriously or minutely damaged by the nanoparticles. In order to improve targeted GBM therapy in an in vivo model, Lilianne Beola et al. [93] investigated a multifunctional lipid magnetic nanocore loaded with TMZ (Ang-TMZ-LMNV) and functionalized with the peptide angiopep-2. Magnetic hyperthermia, which occurs when exposed to alternating magnetic fields (AMF), complements chemotherapeutic drugs. Research conducted on in situ human U-87 MG-Luc2 tumors in nude mice has demonstrated that Ang-TMZ-LMNVs can be administered locally, accumulate and remain in tumors without entering healthy tissues, effectively inhibit tumor invasion and proliferation, and, when combined with AMF stimulation, significantly increase median survival.

#### 3.3.5. Carbon Nanomaterials (CNs)

Carbon nanomaterials are extensively utilized in the biomedical field and have emerged as a focal point for drug delivery carriers [94] due to their distinctive structure, stable properties, ease of modification, and antimicrobial activity [95]. Graphene consists of a layered sheet of two-dimensional carbon atoms arranged in a single hexagonal honeycomb structure with sp2 hybridization [96]. It is also fully waterproof and exceptionally durable, and can be made into optically transparent and biodegradable materials. These inherent properties have the potential to minimize the risk of toxicity while maintaining the integrity of medicinal substances. At the same time, the ability to functionalize the structure of carbon nanoparticles must also be studied in depth in light of any direct and indirect toxicity these nanoparticles can acquire. The main categories include graphene oxide (GO), nanoribbon graphene, oxide nanoribbon graphene, and quantum dot graphene [97]. GO, a compound rich in ROS groups which is an oxygenated derivative of graphene, has become a research hotspot in nanocarriers in recent years due to its excellent thermal stability, robust mechanical strength, and high electronic conductivity [98]. At present, the process through which carbon materials cross the BBB is not fully understood, although RMT is widely acknowledged as the primary pathway. Carbon dots (CDs) modified with transferrin and bound to DOX and temozolomide have higher binding capacity to U87 glioma cells compared to unmodified CDs. The other possible mechanism is passive diffusion. Tight binding within the BBB presents a gap of 4–6 nm [99], suggesting that nanomaterials such as 4 nm diameter CDs and carbon nanoscale particles may potentially traverse the barrier by wedging into the intercellular space between ECs and the BBB. Furthermore, the capacity to penetrate the BBB can be increased by utilizing electrical charges. Functionalized CNTs, among them especially amino-functionalized MWCNTs, are widely used in the fields of bioengineering, medical diagnostics, and therapeutics due to their advantages, such as good aqueous dispersion, high drug loading, and long circulation time in the body. You et al. [100] designed and synthesized a BBB-transpenetrating peptide transcriptional activator (TAT)-coupled oxaliplatin (OXA), a cancer-targeting molecule biotin (B), and PEI-functionalized MWCNTs, TBCNT@OXA, which can be used for precise in situ treatment of gliomas. In this study, TAT-PEI-biotin copolymers were modified on MWCNTs by means of a condensation reaction, and OXA was chosen as the model drug. The hCMEC/C6 co-culture model was employed to mimic the BBB in vitro, and it demonstrated that TBCNT@OXA is able to penetrate the BBB. This nanosystem not only reduces the toxicity of the drug, but also enhances the penetration and targeting of the BBB, and it has the therapeutic potential for CNS disease.

#### 3.3.6. Shuttle Peptide

Shuttle peptides are becoming more widely acknowledged for their affordability, minimal immune response, and wide range of chemical options, including facile chemical alteration. They allow many kinds of goods to cross the BBB, for example, small molecules, genetic materials, proteins, and nanoparticles. A variety of shuttle peptides targeting the BBB have been created to aid in the transportation of nanoparticles, leading to improved distribution throughout the CNS.

Cell-penetrating peptides (CPPs) possess the capability to carry various therapeutic payloads into cells and serve as carriers for transporting drugs targeted specifically for the brain through the BBB. CPPs are short peptides which are typically sufficient to penetrate cell membranes [101], and they have been utilized as a drug delivery vehicle that can cross a variety of biological barriers [102], which includes the BBB [103].

The small peptides may enter the CNS via carrier-mediated transport (CMT), which involves the inflow and outflow of molecules through membrane solute carrier (SLC) proteins and a protein superfamily, which can transport organic solutes, neurotransmitters, and drugs across the BBB [104]. An SLC membrane protein, Peptide Transport System-1, was identified by Kastin and Banks. It transports small peptides with N-terminal tyrosine [105]. Another SLC, known as Organic Anion-Transporting Polypeptide (OATP), plays a role in transporting drugs and endogenous substances, such as neuropeptides, across the BBB [106]. The common characteristic of these transporters is that each transporter has a ligand with a similar structure, and the transportation can be either unidirectional or bidirectional [107]. Peptides can interact with ECs through membrane transporter proteins, leading to changes in transporter function and subsequently altering the permeability of the BBB. Furthermore, through their interactions with permeability glycoprotein-1 (P-gp), a transmembrane protein present in the inner endothelium of brain capillaries, peptides control the permeability of the BBB. This protein has a role in helping medications travel from the brain to the bloodstream. To transfer methotrexate (MTX) over the BBB and into the brain, Shadmani et al. [108] synthesized 50 nm MSN and attached TAT peptide to MSN as a targeted ligand. In vitro tests showed that this nanocarrier system’s absorption rate by brain tissue was 31.13 times higher than that of free MTX. Compared to free MTX, the rate of triggered apoptosis in U87 cells was nine times higher.

Larger peptides and proteins, including regulatory proteins such as cytokines, transferrin, low-density lipoprotein, ghrelin, leptin, IgG, insulin, and insulin-like growth factors, traverse the BBB via RMT utilizing an RMT system. The ligand binds to the receptor on the ECs’ luminal membrane during this phase. The ligand–receptor combination is then absorbed via endocytosis, which results in cytoplasmic vesicles holding the receptor–ligand combination forming. These vesicles may circulate to the luminal surface, be directed to lysosomes for degradation, or shuttle to albumin membranes, where exocytosis occurs, and release their contents into the brain parenchyma [109].

### 3.4. Adsorption-Mediated Transport

AMT serves as a primary pathway for drugs to penetrate the CNS from the systemic circulation through the BBB, thereby contributing to the development of more targeted DDS facilitated by absorption. AMT is short-lived and non-specific. Therefore, combining drugs and cationic groups to construct nano-delivery particles can increase the chances of drugs passing through the BBB [110]. Endocytosis of cells occurs in two steps: first, NPs adhere to the cell membrane, and then internalize via an energy-dependent pathway [111]. The electrostatic attraction of polycationic materials to the negative charges on the surface of ECs initiates endocytosis [112]. The negative charge present on the lumen and surface of BECs is due to the polarization distribution of carboxyl groups in sialic acid-containing glycoproteins and sulfate groups in heparan sulfate proteoglycans on the plasma membrane under a normal physiological environment [63,112].

**Figure 4:**
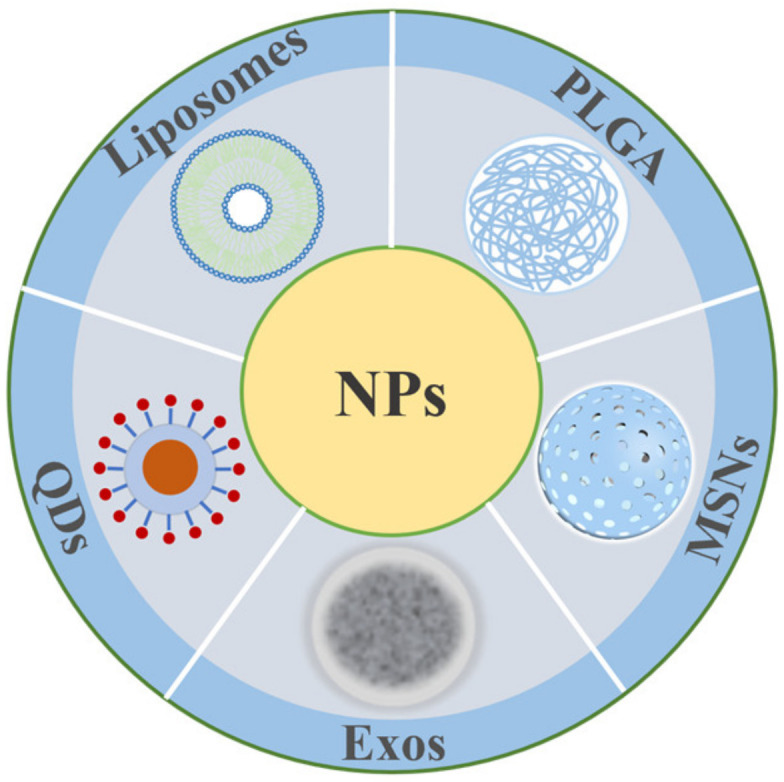
Various types of nanoparticles.

Over the last three decades, there has been a swift advancement in nano-materials, resulting in the production of numerous nanomaterials and DDS with diverse targeting agents in research facilities globally. Nanoparticle drug delivery particles have gradually achieved the characteristics of small particle size, high biocompatibility, and low biotoxicity. Nanoparticle drug delivery provides the benefits of being non-invasive and cost-effective, as well as leading to improved long-term stability, easy synthesis, high targeting efficiency, and controlled drug release through the BBB. This enables the treatment of significant brain diseases through the crossing of the BBB. Nanoparticle-based drugs can penetrate the BBB through RMT, transporter-mediated transcellular transport, and intracellular phagocytosis [113] (as shown in Figure 4). Among them, drug-laden nanoparticles readily couple to receptor ligands in brain vascular ECs; this provides an efficient route for delivering targeted medication to the brain and treating neurological conditions. Indeed, certain neurological disorders can also impact the TJs between ECs, leading to increased permeability of the BBB. Furthermore, some antibody drugs may disrupt the BBB through intramuscular injection or intravenous infusion of specific medications. Alternatively, the BBB can be temporarily bypassed by direct intracranial administration.

#### 3.4.1. Liposomes

Because of their uncomplicated structure and preparation process, liposomes were one of the earliest types of drug-carrying nanoparticles to be created worldwide. The interface between solid and liquid is created by the hydrophilic end of lipids, while the lipid bilayer facing in the opposite direction is formed by the hydrophobic end. The structural similarity between the lipid bilayer in liposomes and biological membranes indicates that they have excellent biocompatibility and can be easily taken up by tissue cells and used as a platform for drug delivery across the BBB. To efficiently deliver medications to the brain, NPs must have a high level of biocompatibility and biodegradability [114]. Compared with some NPs, such as heavy metal NPs or carbon quantum dots that do not degrade or have potential toxicity, liposomes have better bioavailability, higher drug-target binding affinity, low damage and hypersensitivity to normal tissues, sufficient drug-carrying capacity, and low cytotoxicity [115]. Hence, liposomes are the most commonly utilized and earliest forms of nanomaterials, making them potentially one of the most suitable options for packaging materials in intracerebral drug delivery [116]. Liposomes have demonstrated significant potential as carriers in DDS, offering the possibility of improving drug efficacy while minimizing or eradicating associated side effects. There are three main ways to penetrate through the BBB using liposomes: (1) adsorption-mediated cytokinesis, (2) receptor-mediated endocytosis, and (3) disruption of the BBB by external forces.

The first approach suggests that DDS enters the CNS through AMT. Because ECs have negatively charged plasma membranes [117,118], positively charged liposomes can attach to these membranes and transit across the BBB through AMT.

An alternative method involves utilizing the numerous receptors present on the surface of barrier ECs [119], with an emphasis on employing RMT to aid in drug passage through the BBB to the CNS. In this approach, the liposomes attach to specific ligands, such as antibodies and peptides.

Besides the two strategies mentioned above, another non-invasive approach has been investigated that uses external forces to help liposomes cross the BBB. In this strategy, although not preferred, the BBB can be transiently disrupted by the stimulation of an external force so that drugs can directly enter the CNS through the BBB.

Treatments for neurological conditions, including stroke, brain tumors, and dementia, may significantly benefit from protein encoded by mRNA replacement and gene-editing therapies facilitated by lipid nanoparticles (LNPs). Delivering LNPs across the BBB following systemic administration, however, is still not well understood [120]. By inserting the liposome into the insulin-like growth factor-1 receptor (IGF1R), Yang et al. [121] created a brain-targeting liposome that transfers salvianolic acid A (SAA) across the BBB. The findings demonstrated the ability of IGF1R-targeted liposomes loaded with SAA (Lipo/SAA) to transfer SAA into the brain, accumulate in brain tissue, and release SAA continuously for a number of hours. Administration of IGF1R-targeted Lipo/SAA was considerably more effective than no-targeted SAA in reducing infarct size and neuronal damage, improving neurological function, and inhibiting cerebral inflammation.

#### 3.4.2. Polylactic Acid-Glycolic Acid

Since the mid-1990s, polylactic acid–glycolic acid (PLGA) has taken its place among many nanomaterials due to its favorable biocompatibility and biodegradability, making it an excellent candidate for drug delivery [122]. PLGA is produced by combining lactic acid and glycolic acid in specific ratios through copolymerization, and it possesses the capability to undergo controlled degradation. Lactic acid, an intermediate product of PLGA, is a normal metabolite in the body, and the final degradation products are H_2_O and CO_2_. As a result, PLGA has been widely utilized for the preparation of microspheres, nanoparticles, implants, and thin-film agents [123]. In the medical field, PLGA NPs are widely used as drug delivery carriers to carry proteins [124], peptides [125], microbial DNA [126], bacterial or viral DNA [127], and a variety of anticancer drugs [128]. Reagents such as surfactants or polymers are commonly employed to modify the surface of PLGA NPs or to covalently attach targeting ligands to PLGA NPs in order to enhance their ability to penetrate the BBB.

Surface-modified PLGA/PLA NPs were effective in enhancing the permeability of the BBB, but it remains unclear whether their early-stage enhancement of the BBB is sufficiently adequate. Shona Kaya et al. [129] designed a ligand called DAS peptide (NH_2_-GGGGSGCLRVGGRrRrRr-COOH), which has an affinity for the α7 nicotinic acetylcholine receptor (α-7 nAChR) that is present on the second surface of BMECs. The team encapsulated Fitc-dextran, a large hydrophilic molecule (70,000 Da), in PLGA NPs and coupled the DAS peptide to the surface of the NPs, which facilitates the transport of loaded NPs to the BBB via RMT. DAS-coupled PLGA NPs have been identified as an effective vehicle for transporting and releasing large hydrophilic compounds across the BBB via RMT. Gina et al. [130] designed the surfaces of NPs by conjugating Ang-2 directly to polymers. Nanopreparation, or post-preparative anchoring by incorporating thiol-containing cysteine into Ang-2 and conjugating it with maleimide-containing poly (ethylenediamine lactate), was then performed. Next, the amount of Ang-2 bound to the nanoparticles was quantified, and its in vivo capacity to cross the BBB and enter the brain parenchyma was evaluated. Preliminary studies using fluorescence and confocal microscopy showed that PLGA NPs targeted for the brain successfully crossed the BBB.

#### 3.4.3. Quantum Dots

QDs are an innovative type of nanomaterials in the semiconductor category. They are also referred to as nanocrystals, which are typically less than 100 nm in size [131] and were first synthesized in 1981, with good biocompatibility and low tissue toxicity [132]. They have a small size and excellent optical stability and quantum yields, so QDs show promising potential for applications in biomedical imaging and nanotherapy [116]. It has been shown that specific active groups bound to the surface of QDs can realize targeted delivery to the brain [133]. Carbon quantum dots (C-QDs) can reduce the deposition of Aβ [134], indicating that quantum dot nanoparticles may have a role in the treatment of AD [135]. Notably, the use of QDs could potentially cause immune cells to aggregate, leading to an inflammatory response. There is ongoing debate about the potential toxicity of QDs, as they have the ability to stimulate excessive production of reactive oxygen species (ROS) and potentially contribute to mitochondrial dysfunction [136]. Recent animal studies have also demonstrated that QDs can accumulate in the brain during their primary exposure pathway, causing neuroinflammation and thus posing a potential threat to the brain [137]. Different from other traditional QDs, CDs have good biocompatibility and low toxicity because they contain no metal elements [138]. Considering the biological properties and importance of the brain, carbon QDs may be a more suitable material. Multiple research studies have suggested that the compact dimensions of CDs, their polymer-based core structure, and the diverse functional groups present on their surface [139] play a role in their capacity to attach to drug molecules for specific drug release [140,141]. Furthermore, CDs generally demonstrate a high level of photoluminescence (PL), which can be used to monitor the movement of CDs through the BBB [142]. The photoluminescence quantum yield of CDs produced by different synthetic methods varies, with the highest recorded value being 93.3% [143]. In 2021, Emel Kirbas Cilingir et al. [142] synthesized metformin-derived carbon dots (Met-CDs) using a microwave-assisted method. The cytotoxicity studies revealed that Met-CD exhibited minimal toxicity and exceptional biocompatibility towards both non-tumor and tumor cell lines, suggesting that Met-CD is an outstanding candidate for use in live-cell bioimaging studies. In addition, the results of the bioimaging studies demonstrated the ability of Met-CD to cross cell membranes and disperse throughout cellular structures, including nuclei and mitochondria. Finally, a zebrafish study has also demonstrated that Met-CD can cross the BBB without any other ligand. AgAuSe QDs coated with neural stem cell (NSC) membrane were created by Huang et al. [144] to bind the rabies virus glycoprotein (RVG) peptide, therefore reducing AgAuSe QD toxicity and improving its ability to penetrate the blood–brain barrier and target nerve cells. AgAuSe QD can be used in vivo to track and assess how well nanomaterial preparations target nerve cells, penetrate the blood–brain barrier, and circulate blood.

#### 3.4.4. Mesoporous Silica Nanoparticles

MSNs, with their mesoporous structure, hold tremendous potential as nanomaterials across various applications and have garnered considerable attention in the fields of biochemistry and diverse areas of medicine. Monodisperse silica microspheres can easily be functionalized, and their mesoporous nature allows them to adapt to a wide range of therapeutic treatments, as well as provides numerous benefits for the delivery of targeted medications [116]. Among them, monodisperse silica nanomicrospheres are inorganic materials, but they can achieve functionality through conjugation with other inorganic materials while modifying their surface functional groups. By preparing monodisperse silica in conjunction with lactoferrin and modifying it with polyethylene glycol (PEG) [145], it has been found that monodisperse silica can effectively cross the BBB, and it has good stability and biological activity. Monodisperse silica microspheres are biocompatible, and a variety of cell lines are insensitive to oxidative stress induced by them. However, high concentrations of monodisperse silica microspheres can be cytotoxic [146]. Zhang et al. [147] prepared an NP called TMZ/CQ@MSN-RGD by coating with PDA and connecting with arginine–glycine–aspartic (RGD), which improved drug delivery efficiency and enhanced anti-tumor effects. Zhang et al. effectively loaded TMZ and chloroquine into MSNs. According to the study, RGD-MSNs accumulated more in tumor and cell models. Paclitaxel was loaded onto manganese-doped mesoporous silica nanoparticles by Liu et al. [148] through adsorption, allowing the drug to evade the blood–brain barrier and build up in tumors. Furthermore, the surface characteristics of the nanoparticles were altered to become hydrophilic and electrically neutral in order to increase the rate of medication absorption when taken orally. This study offers fresh concepts and approaches for the creation of medications taken orally and the treatment of tumors.

#### 3.4.5. Exosomes

Extracellular vesicles with lipid membranes that have a diameter of less than or equal to 100 nm are known as exosomes. Drug delivery in the brain is complex due to the complex structure of the BBB, and innovative technologies are needed to improve drug delivery (invasive or non-invasive) [149]. Because exosomes have distinct physicochemical properties and are small enough to operate at the nanoscale, they could be a useful tool in solving the aforementioned issues. Exosomes can also swiftly infiltrate cells through interactions with specific cell membrane proteins. As a result, exosomes are thought to be the natural chemical reagents’ most effective carrier [150]. Due to its remarkable properties, including targeted biological distribution of the drug, increased drug load, and improved half-life, it can be used as an effective nanocarrier for TMZ and flavonoids in gliomas [151]. The use of exosomes to deliver chemotherapy drugs has been shown to improve the efficacy of cancer treatment. For example, exosomes containing paclitaxel can be used to treat pancreatic, lung, and prostate cancers [152]. Iyaswamy et al. [153] created an innovative exosome-based tailored medication delivery system. The targeted distribution of Corynoxine-B is achieved by the FE65-engineered HT22 neuron-derived exosome (FE65-EXO) loaded with Corynoxine-B (FE65-EXO-B), which also hijacks signal transduction and prevents the normal interaction between Fe65 and amyloid beta precursor proteins. In 5xFAD mice, Fe65-EXO-B reduces learning and memory deficits and efficiently stimulates the establishment of synaptic plasticity between hippocampus neurons in Alzheimer’s disease. In order to deliver DOX for glioblastoma therapy, Wang et al. [154] developed Pep2, a functional oligopeptide with continuous lysine and cysteine residues, to be included in exosomes generated from BV2 murine microglia (Pep2-Exos-DOX). Pep2-Exos-DOX demonstrated anti-glioblastoma action and effective brain targeting without causing appreciable harm. To improve targeting, Wang et al. [154] created and synthesized three useful oligopeptides to combine with exosomes. Disulfide bonds on cysteine residues in these peptides help improve DOX inclusion, and when large amounts of glutathione (GSH) are found in tumors, disulfide bond breaking takes place, releasing DOX from exosomes. This exosome delivery system has been shown to have good brain-targeting ability and anti-glioblastoma activity in both in vitro and in vivo tests, with minimal observable biological damage.

#### 3.4.6. Other NPs

Akash Ashokan et al. [155] postulated that it may be highly advantageous to create a BBB NP that is dispersed at the main tumor site and across any subsequent brain tumors. They describe a straightforward targeting approach to target primary breast and secondary brain tumors using a single NP platform. These devices’ ability to target and distribute hyperpolarized mitochondrial membranes to extracranial primary tumor locations in addition to brain cancers is made possible by their ability to penetrate the BBB and target mitochondria. They present a proof-of-concept notion to offset increased metabolic flexibility in BMS by lowering oxidative phosphorylation (OXPHOS) and glycolysis, two important energy sources, by using this combination of two anatomically distributed NPS loaded with therapeutic medicines. Through additional testing, they also showed that prechemotherapy drugs, when combined with NP loaded with inhibitors of pyruvate dehydrogenase kinase 1, decrease the effectiveness of OXPHOS and glycolysis. The ultimate findings demonstrate that this combination therapy strategy leverages tactics for mitochondrial genome targeting in order to circumvent the mechanism of chemotherapeutic resistance based on DNA repair.

Organ-on-chips have been shown to be reliable and effective in screening for highly effective DDS by Jeong-Won Choi et al. [156]. They achieved this by comparing the organ-on-chips’ performance to that of a traditional transwell, both of which are intended to replicate the BBB. When compared to bacteria found using the transwell method, BBB nano-shuttle bacteria found by BBB chip-based screening demonstrated better function in vivo. The BBB chip’s capacity to identify molecular shuttles that effectively utilize the endothelial calyx boosts the effectiveness of transendothelial transport. According to their research, organ-on-chip technology shows great promise for furthering the study of vascular-targeting DDS since it can faithfully replicate molecular transport inside the endothelium system.

### 3.5. Physical Method

Based on the physical consequences of employing NPs, there are a few popular strategies for enhancing tissue barrier permeability (Table 1).

## 4. Conclusions

With its low permeability and strong selective hindrance, the BBB serves as a dynamic barrier that separates peripheral blood from neuronal cells in the brain and spinal cord, which is essential for preserving CNS homeostasis. TJs are a complex cell line that has regulatory effects. Because of the existence of TJs in the BBB, all macromolecules and 98% of small molecule drugs have difficulty entering through passive diffusion. Therefore, the BBB is the main obstacle to the administration of certain neurodegenerative diseases. A thorough comprehension of the mechanism underlying BBB breakthrough is crucial for the management of cerebrovascular diseases and the diagnosis of other CNS disorders.

This article focuses on analyzing four different approaches to crossing the BBB, which include passive diffusion, RMT, AMT, and CMT. Small lipophilic drugs can pass through the BBB by passive diffusion, whereas the transportation of many endogenous macromolecules into the brain depends on RMT; CMT does not require energy, but has saturability and high specificity, and some inorganic nanoparticles and penetrating peptides can pass through BBB in this way; and AMT is transient and non-specific, with four types of nanoparticles that can pass through the BBB in this way being presented here: liposomes, polyactive acid glycolic acid, quantum dots, and mesoporous silica nanoparticles. However, I believe that there is still a significant amount of work that can be performed in the future, such as exploring more nanomaterials and coupling them with drugs to enhance the efficiency of drug penetration into BBB. Besides the delivery of drugs, there is combinatorial delivery of other agents (e.g., imaging probes and biosensors), which is expected to improve efficacy through the fabrication of therapeutic nanoplatforms. Furthermore, because of advancements in medical science, treating disorders of the central nervous system no longer requires reliance on a single technology. Rather, a variety of tactics are employed to support complementary approaches to integrated diagnosis and therapy. However, research on many treatments is still in its infancy, and further studies are required in order to fully understand each treatment’s mechanism of action and practical applications.

## Figures and Tables

**Table 1:** Physical methods of crossing the BBB.

Method	Content	Reference
low-intensity pulsed ultrasound (LIPU) mediated transport	Intravenous microvesicles (MB) and LIPU were utilized to break down the BBB and raise the levels of PD-1-blocking antibody (aPD-1) and liposomal adriamycin.	[157]
	Intravascular MBs in conjunction with MRI-guided targeted ultrasound activated the BBB. The findings demonstrated that MRI-guided combination therapy produced accurate and short-term BBB, turning on in the targeted hippocampus and internal entorhinal cortex, which fully recovered to normalcy in less than 24 h.	[158]
	The combination of FUS-MB with claudin-5 adhesive maximizes the transport of medication to the brain. The permeability of the BBB increases when the two types are combined.	[159]
magnetic-field-enhanced permeation	A magnetoelectric nanoparticle for glioma-targeted therapy that has a pegylated IP peptide surface coating (CBPI), a piezoelectric shell made of BaTiO, and a magnetic core made of CoFeO. Through magnetoelectric effects, the nanoparticle down-regulates the BBB’s TJ protein, working in concert with IP peptides to increase BBB permeability and target gliomas.	[160]
electromagnetic field	In a rat model, after 20 min of tDCS treatment at 0.1–1.0 mA, the increase in membrane permeability lasted for about 20 min, rather than immediately returning to normal.	[161]
electroporation	The proteomic cargo of tumor-derived extracellular vesicles, TDEVs, is markedly altered by high-frequency irreversible electroperforation (H-FIRE) ablation of gliomas. This increases the permeability of the BBB endothelium and increases the internalization of TDEVs by brain endothelial cells.	[162]
